# Association of Estimated Insulin Resistance with N-Terminal B-Type Natriuretic Peptide Concentration in Men with Metabolic Syndrome

**DOI:** 10.1155/2019/8571795

**Published:** 2019-12-18

**Authors:** Paweł Krzesiński, Wiesław Piechota, Katarzyna Piotrowicz, Grzegorz Gielerak, Agnieszka Woźniak-Kosek

**Affiliations:** ^1^Department of Cardiology and Internal Diseases, Military Institute of Medicine, Szaserów Street 128, 04-141 Warsaw, Poland; ^2^Department of Laboratory Diagnostics, Military Institute of Medicine, Szaserów Street 128, 04-141 Warsaw, Poland

## Abstract

**Background:**

The diagnostic and prognostic role of N-terminal pro-B-type natriuretic peptide (NT-proBNP) in heart failure is well established. However, additional factors may influence its concentration. One of them is obesity, which in general is accompanied by reduced NT-proBNP levels. However, specific data concerning metabolic syndrome (MS) are equivocal. The aim of the present study was to evaluate the association of NT-proBNP with estimated insulin resistance (eIR) in men with MS.

**Methods:**

In 86 male patients with MS (78 of them hypertensive), blood pressure, anthropometric measures, NT-proBNP, creatinine, glucose, and insulin were assessed and eIR was calculated using homeostatic model assessment (HOMA-IR).

**Results:**

Both eIR and age were independently associated with NT-proBNP concentrations (*b* = 0.2248, *p*=0.019; *b* = 0.0102, *p*=0.049, respectively). Blood pressure, anthropometric measures, and eGFR were not correlated with NT-proBNP. Patients without eIR had higher NT-proBNP than those with eIR (32.2 ± 26.4 vs 21.4 ± 25.4 pg/mL, *p*=0.014). The difference was even higher in the younger subgroup of patients reaching nearly 50%.

**Conclusions:**

Insulin resistance and, to a lesser degree, age were associated with NT-proBNP levels in men with MS. In younger subjects with eIR, mean NT-proBNP level was lower than in corresponding healthy age males.

## 1. Introduction

Amino-terminal pro-B-type natriuretic peptide (NT-proBNP) is a neurohormone synthesized in the cardiac ventricles in response to increased wall tension and stretching. Its diagnostic and prognostic utility in heart failure is well established [1]. It does not have natriuretic activity as equimolar BNP but is more stable in circulation. Factors which may influence the concentration of natriuretic peptides in blood, except heart conditions, include age, body mass index (BMI), hemoglobin level, and kidney function [2]. In population studies, concentration of NT-proBNP was, in general, lower in obese subjects compared to lean ones [3]. This correlation may to some extent compromise the diagnostic and prognostic value of NT-proBNP [4] unless a correcting factor is taken into consideration. Most of the data on associations between obesity and natriuretic peptides come from population studies, whereas studies focused solely on metabolic syndrome (MS) are scarce. Data on NT-proBNP concentration in MS, in which obesity is the main but not the only criterion, are equivocal ranging from reduced [2, 3, 5], through normal [6], to elevated [7]. The pathophysiology of the interdependence of obesity and natriuretic peptides in MS is not quite clear, and insulin resistance (IR) is implicated as a possible cause [8, 9].

The aim of the present study was to evaluate the association of NT-proBNP with estimated insulin resistance (eIR) in men with MS. The secondary aim was to compare NT-proBNP levels in men with and without eIR, also with reference to age and NT-proBNP ranges assumed for healthy ones.

## 2. Methods

### 2.1. Study Group

The study included 86 male patients with MS diagnosed according to the IDF criteria [10] at the Military Institute of Medicine in Warsaw, Poland. The patients had no history of diabetes, chronic kidney disease, thyroid dysfunction, or cardiovascular disease (except arterial hypertension). Anthropometric parameters (waist circumference (WC), BMI, and waist/hip ratio (WHR) were measured according to the standard methods [11]. Office blood pressure (SBP, systolic blood pressure and DBP, diastolic blood pressure) measurements were performed according to the 2017 ACC/AHA Hypertension Guidelines [12]. The study was approved by the local ethics committee (no. 44/WIM/2010). Each patient signed the written informed consent to participate in the study.

### 2.2. Laboratory Tests

After an overnight fast, blood samples were collected by venipuncture into glass tubes (sample no 1) without anticoagulant and tubes containing K3EDTA (sample no 2). Samples no 1, after clotting, were centrifuged at 2000*g* for 15 minutes at the temperature of 4°C to obtain serum. Samples no 2 were used for glycated hemoglobin (HbA1C) determination. Fasting glucose (FG), total cholesterol (TC), low-density lipoprotein cholesterol (LDL-C), high-density lipoprotein cholesterol (HDL-C), and triglycerides (TG) were determined in serum by standard methods. NT-proBNP and insulin were determined by the electrochemiluminescence immunoassay with a Roche Cobas 6000 analyzer. Analytical sensitivity of NT-proBNP test was 5 pg/ml, functional sensitivity was 50 ng/ml, and precision (reproducibility) as coefficient of variation, CV, was 3.1% at the level of 46 pg/ml.

Insulin resistance was quantified according to HOMA, homeostatic model assessment, proposed by Mathius [13] using the following equation: HOMA-IR = [insulin (*μ*U/mL) × glucose (mg/dl)]/405. Cutoff for insulin resistance was adopted as an upper limit of 3 quartile of HOMA-IR, equal to 3.4, established for Polish population by Szurkowska et al. [14]. Estimated glomerular filtration rate (eGFR) was estimated according to the MDRD formula [15].

### 2.3. Statistical Analysis

Statistical analyses were performed with Statistica for Windows, Release 12 (StatSoft, Inc, Tulsa, OK, USA). Data were expressed as means or standard deviations. Mann–Whitney *U* test was used to assess differences between subgroups of patients. In addition, logarithmic transformation was applied for positively skewed data and afterwards *t*-test was used for comparison of means. Spearman correlation coefficients were calculated for selected variables. Multiple linear regression was used to assess factors independently associated with NT-proBNP. Due to highly skewed frequency distribution of NT-proBNP concentrations (median value 18.5 pg/ml and mean 27.9 pg/ml), mostly nonparametric tests were used to assess correlations and significance of differences between subgroups of patients. Logarithmic transformation of the NT-proBNP levels was also used to compare groups with and without insulin resistance. A two-sided *p* value <0.05 was considered significant.

## 3. Results

### 3.1. Basic Characteristics

Arterial hypertension was found in 78 patients, hypertriglyceridemia occurred in 65 patients, and low HDL-C in 43 patients. All patients presented nonimpaired left ventricular systolic function (mean left ventricular ejection fraction 62.9 ± 3.7%, range 56–71%). None fulfilled the criteria for heart failure diagnosis. More than a half of the patients were treated with statins (*n* = 58) and angiotensin-converting enzyme inhibitors (*n* = 52), about one third with diuretics (*n* = 31) and beta-blockers (*n* = 27), and about a quarter with calcium blockers (*n* = 27) and angiotensin receptor blockers (*n* = 20). Fibrates were prescribed in 13 and acetylsalicylic acid in 5 patients. No antidiabetic medicaments were used.

Clinical and biochemical characteristics of the study group are presented in Table 1.

### 3.2. Correlations between NT-proBNP and Other Variables

None of the anthropometric measures (WC, BMI, and WHI) was associated with NT-proBNP concentrations (Table 2). The same applied to SBP, DBP, and eGFR. Similarly to NT-proBNP, SBP and DBP were not correlated with WC, BMI, and WHR (*p* > 0.4, detailed data not shown). NT-proBNP levels were weakly but significantly correlated with age and HOMA-IR, according to Spearman's correlation analysis. We found negative correlation between insulin and NT-proBNP, but it did not reach statistical significance (*p*=0.099).

Both insulin resistance and age were independently associated with NT-proBNP concentrations in the multiple linear regression model (Table 3).

### 3.3. NT-proBNP Concentrations Dependence of Insulin Resistance and Age

**Figure 1:**
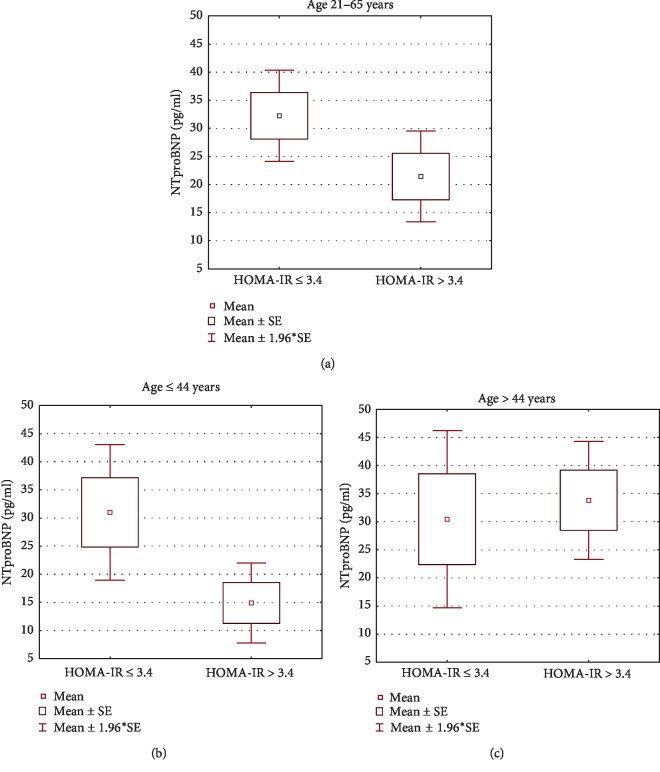
The distribution of NT-proBNP values in relation to HOMA-IR for all subjects (a), subjects aged ≤ 44 years (b), and subjects aged > 44 years (c).

Mean NT-proBNP level (log scale) was significantly lower in the eIR subgroup compared with patients without eIR (log [NT-proBNP]: 1.105 vs 1.338, *p*=0.017), with no coexisting difference in age (42.7 ± 9.6 vs 43.0 ± 9.6 years, *p*=0.895). The difference was greater in the younger subgroup of patients (≤44 years), Table 4 and Figure 1. Lower mean values of NT-proBNP in the subgroup with eIR were, to great extent, due to increased frequency of extremely low values of NT-proBNP of 5 pg/ml corresponding to the limit of detection (analytical sensitivity of the test). Such results occurred in approximately 28% of all patients and in 42% of eIR patients. Furthermore, 60% of patients with eIR revealed NT-proBNP below the median value (18.5 pg/ml), in comparison to 40% of those without eIR.

### 3.4. Comparison of NT-proBNP Concentrations in Men with MS with reference to Healthy Subjects

The comparison of concentrations of NT-proBNP in our patients with reference values obtained from a large sample of healthy male subjects reported by Hess et al. [16] is presented in Table 5. Mean NT-proBNP concentration in younger subjects (23.1 pg/ml) appeared to be somewhat lower than that in the reference group (27.7 pg/ml), but wide confidence limit (CL) of the mean did not allow to draw a conclusion that the difference was statistically significant. However, when we compare mean NT-proBNP (14.9 pg/ml) in the subgroup of 22 younger eIR subjects (Table 4) with mean NT-proBNP (27.7 pg/ml) in the corresponding reference group (Table 5), the difference becomes more pronounced.

## 4. Discussion

In our study, we revealed the interrelation between NT-proBNP and IR in MS patients with no history of diabetes or cardiovascular disease other than hypertension. In this relatively homogenous patients group, only eIR and age were correlated with NT-proBNP. No relevant relationship was found for BP and anthropometric indices. Moreover, we showed that the interdependence of eIR and low level of NT-proBNP was more pronounced in younger subjects.

Performing our analysis in such a carefully selected group, we have eliminated some potentially affecting factors. We confirmed no association between NT-proBNP and renal function among MS patients because chronic renal disease was an exclusion criterion in our study. No correlation between NT-proBNP and blood pressure, allowed excluding a significant difference in cardiac load as a potential bias.

The lack of the association between anthropometric measures and NT-proBNP in our patients was in agreement with the report of Chang et al. [9], who in 74 hypertensive patients (60% with MS) did not find a correlation of NT-proBNP with BMI, WC, and body fat mass. Similarly, in a large population study, which included subjects without overweight/obesity and with MS, no significant correlation was found between NT-proBNP and either BMI or WC [17]. In another study, abdominal obesity was associated with lower NT-proBNP levels in females, but not in males [18]. Yet, another study indicated that only BMI and not WC was negatively but weakly associated with NT-proBNP concentrations in MS patients [19]. Our findings suggest that lack of IR evaluation may be responsible for these discrepancies.

When we stratified our patients according to eIR, the concentration of NT-proBNP was significantly lower in eIR subjects. The important role of IR in determining the relation between obesity and MS with NT-proBNP levels was proven by Baldassarre et al. [17], who observed that inverse association between BMI and NT-proBNP in general population was no longer significant after the inclusion of insulin resistance (HOMA-IR) into the logistic regression model. An earlier large ambulatory population study also indicated that IR is responsible for 10–30% decrease of NT-proBNP levels in both obese and normal weight subjects [20]. Our eIR patients had 33% lower NT-proBNP average level than those without eIR. Our results resemble partially those obtained by Fu et al. [21] who found that serum NT-proBNP concentrations in the individuals with IR were lower than in those without IR, but the presence of MS did not matter. We also noted that the difference was even larger in younger patients. It suggests that with aging the relation of NT-proBNP with IR may become overlapped by other factors.

Pathophysiological associations of natriuretic peptides with IR are not fully explained. Insulin increases the expression of natriuretic peptides clearance receptors (NPRC) in adipose tissue and might suppress circulating natriuretic peptides via upregulation of NPRC expression [8]. In addition, expression of neutral endopeptidase degrading natriuretic peptides (neprilysin) in adipose tissue is increased in obesity [22] and probably in IR [23]. It seems that these mechanisms cannot be directly applied to explain NT-proBNP decrease in IR as it is mainly cleared by the kidneys [24] and not catabolized by the abovementioned pathways. Instead, an alternative mechanism is proposed, namely, decreased natriuretic peptides release from the heart, rather than clearance by adipocytes, explains the association of obesity and IR with decreased levels of natriuretic peptides [25, 26]. This explanation is plausible as BNP and NT-proBNP are produced in equimolar quantities, and their plasma concentrations are highly correlated [27]. It is suggested that lower levels of natriuretic peptides could be a consequence rather than a cause of IR [28], but this opinion is not firmly established [29].

### 4.1. Clinical Implications

These phenomena may be clinically relevant. Relative natriuretic deficiency, so-called natriuretic handicap, in conditions with high frequency of IR, i.e., obesity and diabetes type 2, may be a risk factor for hypertension as the peptides prevent sodium retention and excessive sympathetic tone [30]. Low natriuretic peptide concentrations in the subjects of the longitudinal Malmö Diet and Cancer Study were also predictive of new-onset diabetes [31]. On the contrary, increased concentration of natriuretic peptides may be protective against insulin resistance [32].

NT-proBNP levels below 125 pg/ml are used to exclude heart failure in general population (with high negative predictive value) [33]. In overweight and obese patients with acute dyspnea, relatively lower NT-proBNP levels still maintain their diagnostic and prognostic value [34]. Whether the specific cutoff should be used for obese subjects (with or without MS, with or without IR) for the same purpose may be debatable.

### 4.2. Limitations

The main limitation of our study is a small study group. We also used HOMA-IR to estimate insulin resistance, not euglycaemic hyperinsulinaemic clamps. We should also be aware of within-subject and between-subject NT-proBNP biologic variations, but the differences between insulin-resistant and noninsulin-resistant subjects in our study were greater than the biologic variation reported for healthy men previously [35]. Moreover, our observations are limited to men and cannot be extrapolated to women.

## 5. Conclusions

In males with metabolic syndrome

1. Insulin resistance and, to a lesser degree, age were associated with NT-proBNP levels. No associations between NT-proBNP and BP, anthropometric parameters, and eGFR were found.
2. In younger patients with eIR, mean NT-proBNP level was lower than in healthy males of similar age.

## Figures and Tables

**Table 1:** Clinical and biochemical characteristics of the study group.

Variables	Mean ± SD	Median, range
Age (years)	43.5 ± 9.6	44 (21.0–65.0)
WC (cm)	111.7 ± 8.9	113.0 (93.0–135.0)
BMI (kg/m^2^)	32.43 ± 3.70	31.2 (23.5–42.36)
WHR	1.09 ± 0.05	1.09 (0.95–1.21)
SBP (mmHg)	142 ± 16.4	149 (110–180)
DBP (mmHg)	90.5 ± 10.1	90.0 (70–110)
NT-proBNP (pg/ml)	27.9 ± 27.1	18.5 (5.0–101.1)
TG (mg/dl)	240.5 ± 124.7	210.0 (61.0–653.0)
HDL-C (mg/dl)	41.2 ± 8.7	40.5 (25.0–66.0)
TC (mg/dl)	220.9 ± 48.5	228.0 (107.0–342.0)
LDL-C (mg/dl)	132.2 ± 39.5	134.5 (28.0–234.0)
FG (mg/dl)	100.1 ± 10.0	99.0 (82.0–125.0)
HbA1c (%)	5.81 ± 0.36	5.75 (5.10–6.80)
Creatinine (mg/dl)	0.88 ± 0.12	0.90 (0.60–1.30)
eGFR (ml/min/1,73 m^2^)	102.3 ± 17.2	105.5 (65.0–157.0)
Insulin (*μ*U/ml)	16.31 ± 9.49	13.6 (3.71–55.13)
HOMA-IR	4.10 ± 2.50	3.26 (0.89–13.07)

BMI: body mass index; DBP: diastolic blood pressure; eGFR: estimated glomerular filtration rate; FG: fasting glucose; HbA1C: glycated hemoglobin; HDL-C: high-density lipoprotein cholesterol; HOMA-IR: homeostatic model assessment (insulin resistance); LDL-C: low-density lipoprotein cholesterol; NT-proBNP: amino-terminal pro-B-type natriuretic peptide; SBP: systolic blood pressure; TC: total cholesterol; TG: triglycerides; WC: waist circumference; WHR: waist/hip ratio.

**Table 2:** Correlation (Spearman *R*) of NT-proBNP with anthropometric, BP and laboratory measures.

Variables	NT-proBNP	*p*
Age (years)	0.23	0.035
WC (cm)	0.03	0.807
BMI (kg/m^2^)	−0.05	0.674
WHR	−0.12	0.300
SBP (mmHg)	0.07	0.501
DBP (mmHg)	0.02	0.889
Creatinine (mg/dl)	0.01	0.986
eGFR (ml/min/1.73 m^2^)	−0.09	0.397
HOMA-IR	−0.23	0.045
Insulin	−0.19	0.099

BMI: body mass index; DBP: diastolic blood pressure; eGFR: estimated glomerular filtration rate; HOMA-IR: homeostatic model assessment (estimated insulin resistance); NT-proBNP: amino-terminal pro-B-type natriuretic peptide; SBP: systolic blood pressure; WC: waist circumference; WHR: waist/hip ratio.

**Table 3:** Parameters affecting NT-proBNP (log NT-proBNP)—multiple linear regression.

Parameter	*b*	*b* standard error	*p*
Constant	0.9012	0.2276	0.0002
HOMA-IR^*∗*^	0.2248	0.0936	0.0188
Age	0.0102	0.0051	0.0487
	*R*	*R* ^2^	
Correlation	0.3447	0.1188	0.0081

^*∗*^Dichotomous predictor: HOMA-IR ≤ 3.4 = 0; HOMA-IR > 3.4 = 1. HOMA-IR: homeostatic model assessment (estimated insulin resistance).

**Table 4:** Comparison of levels of NT-proBNP in the subgroups with and without IR.

Variable	HOMA-IR ≤ 3.4 (without eIR)		HOMA-IR > 3.4 (with eIR)		*p* ^*∗*^
	Mean ± SD (pg/mL)	*N*	Mean ± SD (pg/mL)	*N*	
NT-proBNP age 21–65 years	32.2 ± 26.4	41	21.4 ± 25.4	38	0.014
NT-proBNP age ≤ 44 years	31.0 ± 29.5	23	14.9 ± 16.9	22	0.030
NT-proBNP age > 44 years	33.8 ± 22.6	18	30.4 ± 32.2	16	0.227

^*∗*^ *p*, Mann–Whitney test; HOMA-IR: homeostatic model assessment (estimated insulin resistance); NT-proBNP: amino-terminal pro-B-type natriuretic peptide.

**Table 5:** The comparison of NT-proBNP concentrations in the study group with reference values.

Metabolic syndrome				Percentile	
Age	Mean, CL	SD	Median	95^th^	97.5^th^
21–44	23.1 (15.3–30.7)	25.3	14.3	81.6	86.3
45–54	36.0 (24.4–47.6)	30.1	29.0	97.9	101.1

Reference values [16]					
Age	Mean	SD	Median	95^th^	97.5^th^

18–44	27.7	25.5	20.0	62.9	85.8
45–54	39.0	63.6	21.6	83.9	121

## Data Availability

The data used to support the findings of this study are available from the corresponding author upon request.

## References

[1] Palazzuoli A., Gallotta M., Quatrini I., Nuti R. (2010). Natriuretic peptides (BNP and NT-proBNP): measurement and relevance in heart failure. *Vascular Health and Risk Management*.

[2] Tanaka A., Yoshida H., Kawaguchi A. (2017). N-terminal pro-brain natriuretic peptide and associated factors in the general working population: a baseline survey of the Uranosaki cohort study. *Scientific Reports*.

[3] Zeng Q., Dong S.-Y., Wang M.-L., Li J.-M., Ren C.-L., Gao C.-Q. (2016). Obesity and novel cardiovascular markers in a population without diabetes and cardiovascular disease in China. *Preventive Medicine*.

[4] Mehrotra A. K., Hampole C. V., Goonewardena S. N. (2008). NT-proBNP levels are lower in patients admitted with acute decompensated heart failure that have metabolic syndrome than those without. *Journal of Cardiac Failure*.

[5] Bao Y., Shang X., Zhou L., Hu R., Li Y., Ding W. (2011). Relationship between N-terminal pro-B-type natriuretic peptide levels and metabolic syndrome. *Archives of Medical Science*.

[6] Sezen Y., Baş M., Demirbag R., Yildiz A., Celik H., Aksoy S. (2009). N-terminal pro-brain natriuretic peptide in cases with metabolic syndrome and its relationship with components of metabolic syndrome and left ventricular mass index. *Clinical Biochemistry*.

[7] Bruno G., Barutta F., Landi A. (2015). Levels of N-terminal pro brain natriuretic peptide are enhanced in people with the uncomplicated metabolic syndrome: a case-cohort analysis of the population-based Casale Monferrato study. *Diabetes/Metabolism Research and Reviews*.

[8] Pivovarova O., Gögebakan Ö., Klöting N. (2012). Insulin up-regulates natriuretic peptide clearance receptor expression in the subcutaneous fat depot in obese subjects: a missing link between CVD risk and obesity?. *The Journal of Clinical Endocrinology & Metabolism*.

[9] Chang H.-R., Hsieh J.-C., Chen M. Y.-C. (2014). N-terminal pro-B-type natriuretic peptide is inversely associated with metabolic syndrome in hypertensive patients. *The American Journal of the Medical Sciences*.

[10] Alberti K. G. M., Zimmet P., Shaw J. (2005). The metabolic syndrome-a new worldwide definition. *The Lancet*.

[11] Sánchez-García S., García-Peña C., Duque-López M. X. (2007). Anthropometric measures and nutritional status in a healthy elderly population. *BMC Public Health*.

[12] Carey R. M., Whelton P. K. (2018). The 2017 American College of Cardiology/American Heart Association Hypertension Guideline: a resource for practicing clinicians. *Annals of Internal Medicine*.

[13] Matthews D. R., Hosker J. P., Rudenski A. S., Naylor B. A., Treacher D. F., Turner R. C. (1985). Homeostasis model assessment: insulin resistance and *β*-cell function from fasting plasma glucose and insulin concentrations in man. *Diabetologia*.

[14] Szurkowska M., Szafraniec K., Gilis-Januszewska A., SzybinS˜ki Z. (2005). Insulin resistance indices in population-based study and their predictive value in defining metabolic syndrome. *Epidemiology*.

[15] Levey A. S., Coresh J., Greene T. (2006). Using standardized serum creatinine values in the modification of diet in renal disease study equation for estimating glomerular filtration rate. *Annals of Internal Medicine*.

[16] Hess G., Runkel S., Zdunek D., Hitzler W. E (2005). N-terminal pro-brain natriuretic peptide (NT-proBNP) in healthy blood donors and in patients from general practitioners with and without a diagnosis of cardiac disease. *Clinical Laboratory*.

[17] Baldassarre S., Fragapani S., Panero A. (2017). NTproBNP in insulin-resistance mediated conditions: overweight/obesity, metabolic syndrome and diabetes. The population-based Casale Monferrato Study. *Cardiovascular Diabetology*.

[18] Suthahar N., Meijers W. C., Ho J. E. (2018). Sex-specific associations of obesity and N-terminal pro-B-type natriuretic peptide levels in the general population. *European Journal of Heart Failure*.

[19] Li W.-Y., Chiu F.-C., Chien Y.-F., Lin J.-W., Hwang J.-J. (2011). Association of amino-terminal pro-brain natriuretic peptide with metabolic syndrome. *Internal Medicine*.

[20] Khan A. M., Cheng S., Magnusson M. (2011). Cardiac natriuretic peptides, obesity, and insulin resistance: evidence from two community-based studies. *The Journal of Clinical Endocrinology & Metabolism*.

[21] Fu S., Ping P., Leiming L., Ye P. (2016). Deep analyses of the associations of a series of biomarkers with insulin resistance, metabolic syndrome, and diabetes risk in nondiabetic middle-aged and elderly individuals: results from a Chinese community-based study. *Clinical Interventions in Aging*.

[22] Standeven K. F., Hess K., Carter A. M. (2011). Neprilysin, obesity and the metabolic syndrome. *International Journal of Obesity*.

[23] Schlueter N., de Sterke A., Willmes D. M., Spranger J., Jordan J., Birkenfeld A. L. (2014). Metabolic actions of natriuretic peptides and therapeutic potential in the metabolic syndrome. *Pharmacology & Therapeutics*.

[24] Takase H., Dohi Y. (2014). Kidney function crucially affects B-type natriuretic peptide (BNP), N-terminal proBNP and their relationship. *European Journal of Clinical Investigation*.

[25] Das S. R., Drazner M. H., Dries D. L. (2005). Impact of body mass and body composition on circulating levels of natriuretic peptides. *Circulation*.

[26] Costello-Boerrigter L. C., Burnett J. C. (2009). A new role for the natriuretic peptides. *Journal of the American College of Cardiology*.

[27] Curiati M. N. C., Silvestre O. M., Pires L. J. T. (2013). Comparação entre BNP e NT-proBNP quanto à concordância e quanto à influência das variáveis clínicas e laboratoriais. *Einstein (São Paulo)*.

[28] Halbirk M., Nørrelund H., Møller N., Schmitz O., Bøtker H. E., Wiggers H. (2010). Short-term changes in circulating insulin and free fatty acids affect Nt-pro-BNP levels in heart failure patients. *International Journal of Cardiology*.

[29] Moro C. (2017). Does insulin resistance trigger natriuretic peptide deficiency?. *EBioMedicine*.

[30] Wang T. J., Larson M. G., Levy D. (2004). Impact of obesity on plasma natriuretic peptide levels. *Circulation*.

[31] Magnusson M., Jujic A., Hedblad B. (2012). Low plasma level of atrial natriuretic peptide predicts development of diabetes: the prospective Malmö Diet and cancer study. *The Journal of Clinical Endocrinology & Metabolism*.

[32] Neeland I. J., Winders B. R., Ayers C. R. (2013). Higher natriuretic peptide levels associate with a favorable adipose tissue distribution profile. *Journal of the American College of Cardiology*.

[33] Ponikowski P., Voors A. A., Anker S. D. (2016). 2016 ESC guidelines for the diagnosis and treatment of acute and chronic heart failure: the task force for the diagnosis and treatment of acute and chronic heart failure of the European Society of Cardiology (ESC). Developed with the special contribution of the Heart Failure Association (HFA) of the ESC. *European Journal of Heart Failure*.

[34] Bayes-Genis A., Lloyd-Jones D. M. (2007). Effect of body mass index on diagnostic and prognostic usefulness of amino-terminal pro-brain natriuretic peptide in patients with acute dyspnea. *Archives of Internal Medicine*.

[35] Melzi d’Eril G., Tagnochetti T., Nauti A. (2003). Biological variation of N-terminal pro-brain natriuretic peptide in healthy individuals. *Clinical Chemistry*.

